# Inhibition of angiotensin II type 1 receptor by candesartan reduces tumor growth and ameliorates fibrosis in colorectal cancer

**DOI:** 10.17179/excli2021-3421

**Published:** 2021-05-07

**Authors:** Ehsan Tabatabai, Majid Khazaei, Fereshteh Asgharzadeh, Seyedeh Elnaz Nazari, Neda Shakour, Hamid Fiuji, Aghigh Ziaeemehr, Asma Mostafapour, Mohammad Reza Parizadeh, Mohammad Nouri, Seyed Mahdi Hassanian, Farzin Hadizadeh, Gordon A Ferns, Mohammad Rahmati, Farzad Rahmani, Amir Avan

**Affiliations:** 1 Stem Cell Research Center, Tabriz University of Medical Sciences, Tabriz, Iran; 2 Department of Clinical Biochemistry, Faculty of Medicine, Tabriz University of Medical Sciences, Tabriz, Iran; 3 Department of Physiology, Faculty of Medicine, Mashhad University of Medical Sciences, Mashhad, Iran; 4 Metabolic Syndrome Research Center, Mashhad University of Medical Sciences, Mashhad, Iran; 5 Department of Medicinal Chemistry, School of Pharmacy, Mashhad University of Medical Sciences, Mashhad, Iran; 6 Student Research Committee, Mashhad University of Medical Sciences, Mashhad, Iran; 7 Department of Medical Biochemistry, Faculty of Medicine, Mashhad University of Medical Sciences, Mashhad, Iran; 8 Biotechnology Research Center, Pharmaceutical Technology Institute, Mashhad University of Medical Sciences, Mashhad, Iran; 9 Brighton & Sussex Medical School, Division of Medical Education, Falmer, Brighton, Sussex BN1 9PH, UK; 10 Iranshahr University of Medical Sciences, Iranshahr, Iran; 11 Medical Genetics Research Center, Mashhad University of Medical Sciences, Mashhad, Iran

**Keywords:** Candesartan, colorectal cancer, renin-angiotensin system

## Abstract

Colorectal cancer (CRC) is an important cause of cancer-related mortality. Aberrant activation of the renin-angiotensin system (RAS) is reported to be associated with poor clinical outcomes in patients with CRC. This study was designed to explore the anti-tumor effects of the angiotensin receptor blocker Candesartan either alone or in combination with 5-FU in *in vitro* and *in vivo* models of CRC. The cytotoxic effects of Candesartan were assessed using the MTT assay in two colorectal cancer cell lines (CT-26 and SW-480). To investigate the potential regulatory role of Candesartan on tumor growth, apoptosis, and migration, the expression levels of Cyclin D1, Survivin, MMP3, MMP9, and E-cadherin mRNAs were evaluated. The oxidant/antioxidant balance was also examined by determining the levels of MDA, thiols, SOD, and CAT. We used a xenograft model of colon cancer to investigate the effects of Candesartan alone, or in combination with 5-FU, on tumor growth following histological staining (Hematoxylin & Eosin and Masson trichrome staining) and biochemical studies as well as gene expression analyses by RT-PCR and western blotting. Candesartan suppressed tumor cell proliferation and migration by modulating Cyclin D1, MMP3/9, and E-cadherin. Treatment with Candesartan either alone, or in combination with 5-FU decreased tumor size in the mouse model, and also increased the level of oxidative markers MDA and reduced CAT, SOD, and thiols. Histological evaluation showed that Candesartan increased tumor necrosis, reduced tumor density and attenuated collagen deposition reducing tumor fibrosis in tumor xenograft. Candesartan, an inhibitor of the RAS, when used in combination with 5-FU reduced tumor growth by inhibiting fibrosis and inducing ROS production, supporting further clinical studies on this therapeutic approach for treatment of CRC.

## Introduction

Colorectal cancer (CRC) is one of the most common and aggressive malignancies with a high mortality globally. Despite recent improvements in treatment modalities or identification of novel diagnostic biomarkers, the overall survival of CRC patients treated with standard chemotherapeutic regiments remains extremely poor (Enofe et al., 2020[19]; Wong et al., 2021[49]). Thus, it is necessary to identify novel alternative therapeutics for treatment and management of CRC. 

There is growing evidence suggesting a possible association between cancer incidence and the use of angiotensin II receptor type 1 (ATR1) blockers, that are used in the management of hypertension and cardiovascular disease. ATR1 have been shown to be upregulated in various cancer cells including melanoma, glioblastoma, prostate, renal, bladder, breast, and pancreatic cancer (Miyajima et al., 2002[33]; Du et al., 2012[18]; Renziehausen, 2017[44]; Renziehausen et al., 2019[43]; Afsar et al., 2021[2]; Arjmand et al., 2020[6]; Asgharzadeh et al., 2020[9]). The overexpression of ATR1 was reported to be associated with the malignant features of tumors by inducing angiotensin II. Angiotensin II is the main effector of the renin-angiotensin system, and has a prominent role in renal and vascular homeostasis. Additionally, the renin-angiotensin system has also been shown to regulate various aspects of tumor growth and development including: cell proliferation, angiogenesis, inflammation, and apoptosis. Further studies indicate that angiotensin II exerts its tumorigenic effects through binding to the ATR1 and angiotensin II receptor type II (ATRII) (Childers, 2015[17]; Pei et al., 2017[37]; Ishikane and Takahashi-Yanaga, 2018[25]). 

AT1R blockers, such as candesartan, losartan, and valsartan are commonly used as anti-hypertensive drugs in patients with cardiovascular diseases (Minatoguchi et al., 2013[32]; Abraham et al., 2015[1]). There is growing evidence that suggests that the suppression of angiotensin II receptor signaling by AT1R blockers can inhibit tumor progression in several types of cancers including renal, colon, breast, bladder, and prostate cancer (Piastowska-Ciesielska et al., 2013[39]; Arrieta et al., 2015[7]; Asgharzadeh et al., 2018[10], 2020[9]; Hashemzehi et al., 2021[23][24]). Candesartan has been reported to inhibit cancer cell growth and development in multiple human tumors when used alone or in combination with other anticancer drugs (Molteni et al., 2006[34]; Fan et al., 2016[20]; Ishikane and Takahashi-Yanaga, 2018[25]). In support of these findings, it has been shown that administration of candesartan, prevents metastasis of renal cancer in a rat allograft model (Miyajima et al., 2002[33]). Moreover, treatment with AT1R blockers has been reported to be associated with longer progression free survival and overall survival in patients with advanced pancreatic cancer and colon cancer (Mc Menamin et al., 2012[30]; Nakai et al., 2013[35]). Taken together, these findings suggest an important role of suppressing AT1R in patient with colon cancer and led us to investigate whether an AT1R blocker may inhibit CRC cell growth either alone or in combination with other chemotherapeutic drugs.

Here we investigated the therapeutic potential and the molecular mechanism of actions of candesartan in the CRC progression by using cellular system and animal model, for a better understanding and hence a better management of the disease.

## Material and Methods

### Drugs and chemicals

Candesartan and 5-FU were purchased from Cayman Chemical (Ann Arbor, MI). Roswell Park Memorial Institute (RPMI) 1640 Medium, fetal bovine serum (FBS), penicillin (50 IU/ml) and streptomycin (50 μg/ml) were purchased from Gibco (Gaithersburg, MD). Other laboratory chemicals were obtained from Sigma-Aldrich (Zwijndrecht, The Netherlands).

### Cell culture 

Two different CRC cell lines (CT-26 and SW-480) were used for the experiments. The cells were purchased from Pasteur Institute (Tehran, Iran). Cells were grown in RPMI 1640 medium supplemented with 10 % FBS (Gibco, USA) and 1 % penicillin/streptomycin at 37.0 °C in 5 % CO_2_ humidified air.

### Growth inhibition (MTT) assay

The MTT tetrazolium colorimetry assay was used to evaluate the cytotoxicity of candesartan on the CRC cells. Briefly, the CT-26 and SW-480 cells were seeded in a 96-well plate and treated with different concentrations of candesartan for 24 hours. After cell treatment, 20 μL of the MTT solution (5 mg/ml) was transferred to each well and plates were incubated at 37 °C for 4 hours to investigate the viable cells can produce formazan crystals. Then, the medium was replaced by DMSO (100 μL) to solubilize the formazan crystals formed by living cells. The optical density was then measured at 545-630 nm using a micro plate reader (TECAN, Austria) (Amerizadeh et al., 2018[5]). Mean results were reported from three independent experiments carried out at least in triplicate. 

### Animal experiment

Eight-week-old inbred BALB/c mice were obtained from the Pasteur Institute (Tehran, Iran). The CT-26 cells (2×10^6^ cells) were subcutaneously injected into the right flank of each mouse. Next, the mice were divided into 4 groups: group 1 (control animals), group 2 were treated with CRC standard treatment, 5-FU (5 mg/kg every other day). Mice in group 3 were treated with candesartan (20 μM) and group 4 was treated with a 5-FU/ candesartan combination. Treatment was started approximately 2 weeks after injection when the tumor volume reached 80-100 mm^3^. The animal tumors were carefully monitored and measured every other day. The tumor volume (V) was calculated according to the formula V = AB2/2, where A was the length of the major axis and B was the length of the minor axis. At day 21, mice were sacrificed and colon tumors were collected for further examination by trichrome and hematoxylin and eosin staining methods (Amerizadeh et al., 2018[5]; Hashemzehi et al., 2021[24]). All animal procedures were conducted according to the guidelines established by the Ethics Committee of Mashhad University of Medical Sciences.

### Quantitative Real-Time Polymerase Chain Reaction (qRT-PCR) 

Total RNA was extracted from the treated cells (7×10^5^ cells/well, in 6-well plates) using the RNeasy® mini kit (Qiagen GmbH, Hilden, Germany). Quantification and quality control of total RNA was performed in triplicate by a NanoDrop2000 spectrophotometer (Thermo Fisher Scientific, Inc.). Then, RNAs were reverse-transcribed using the Prime-Script™ RT reagent kit (TaKaRa Holdings, Inc., Kyoto, Japan) (Afshari et al., 2019[3]; Rahmani et al., 2019[40]). Also, quantitative RT-PCR analysis was performed using RealQ Plus 2X MasterMix Green-without Rox™ (Amplicon, Stenhuggervej, Denmark). Next, quantitative RT-PCR was performed using specific primers for Cyclin D1, Survivin, E-cadherin, MMP-3, MMP-9, and GAPDH (Table 1(Tab. 1)) that were obtained from Macrogene (Macrogene Co., Seoul, Korea). The cDNA amplification was performed by using the LightCycler 96 real-time PCR system (Roche Applied Science, Pleasanton, CA, USA). Also, gene expression data were normalized against GAPDH. The 2−ΔΔCt method was used to analyze the relative expression of target genes. The primer sequences (forward and reverse) are listed in Table 1(Tab. 1).

### Quantification of apoptosis

The regulatory effect of candesartan on CRC cell apoptosis was studied by flow cytometric analysis using Annexin V/PI apoptosis assay kit (Cayman Chemical, Ann Arbor, MI) (Ghanaatgar‐Kasbi et al., 2019[21]). In brief, CT-26 and SW-480 cells were exposed to candesartan (20 μM) for 24 hours and the population of apoptotic cells were detected by flow cytometry according to manufacturer's instruction. Each experiment was performed in triplicate.

### Histopathological staining 

The CRC tumor tissues were fixed in formaldehyde, and subsequently embedded into paraffin blocks. The paraffin-embedded blocks were then sectioned to a thickness of approximately 5-7 µm and multiple tissue sections were mounted onto a glass microscope slide. Following de-paraffinization, mounted tissue sections were stained with hematoxylin and eosin and Masson's trichrome and reviewed by at least 2 independent researchers (Rahmani et al., 2020[41]; Soleimani et al., 2020[46]).

### Migration assay

The migration assay was performed as described previously (Hashemzehi et al., 2018[22]). Cells were treated with candesartan and cellular migration was evaluated using the Leica DMI300B (Leica) at different time intervals (0, 16, 26, 46hr) and quantified by Image J software.

### Measurement of oxidative stress markers

To further explore the regulatory effects of candesartan on oxidant/antioxidant balance in tumor tissues, the catalase (CAT), superoxide dismutase (SOD), malondialdehyde (MDA) and total thiol levels were determined in homogenized colon samples as described previously (Asgharzadeh et al., 2021[8]).

### Docking study

The interaction of the anti-cancer drug (Candesartan Cilexetil) with the active site of the target proteins was examined by Molecular Operating Environment docking experiment (MOE, Chemical Computing Group Inc. Montreal, https://www.chemcomp.com/). No previous report was published associate to interaction and inhibition of mentioned target proteins in this article by Candesartan Cilexetil. The chemical structure of Candesartan was drawn using a software program (ChemDraw Ultra 7.0) and its energy minimized by MOE, respectively. Then we downloaded the crystal structure of proteins from the RCSB Protein Data Bank. PDB ID used in this study were 4APJ (for ACE), 5QIN (for TGF-beta type II), 3TZM (for TGF-beta type I), 4ZUD (for AT1), 4G9L (for MMP3), 3D91 (for Renin), 1GKC (for MMP9), 1XOX (for Survivin), 5cvb (for COL1A1), 2O72 (for E-cadherin), 5CTI (for COL1A2), 3KBH (for ACE2), 1ALU (for IL-6), 7JRA (for TNF-α), and 5VZU (for Cyclin D1). The docking procedure was performed using the default settings of the MOE-DOCK. The final docking scores were evaluated using the GBVI/WSA dG scoring function with the Generalized Born solvation model (GBVI) (Shakour et al., 2020[45]). The GBVI/WSA dG is a force field-based scoring function, which estimates the free energy of binding of the ligand from a given pose. The inhibition constant (Ki) was computed through the binding free energy estimated with the GBVI/WSA dG scoring function according to the following equation: ∆G=RTLn(Ki) where R represented the gas constant and T the temperature in kelvin. The Ki was computed from the binding free energy values at a fixed temperature (298 K).

### Statistical analysis

All assays were undertaken in triplicate and verified in at least two independent experiments. All results were expressed as mean ± SD, and analyzed by Student's t-test following by Tukey's multiple comparison tests or Kruskal Wallis tests and Mann Wilcoxon U tests. The statistical significance was considered lower than 0.05. 

## Results

### Candesartan inhibits cell viability

**Figure 1:**
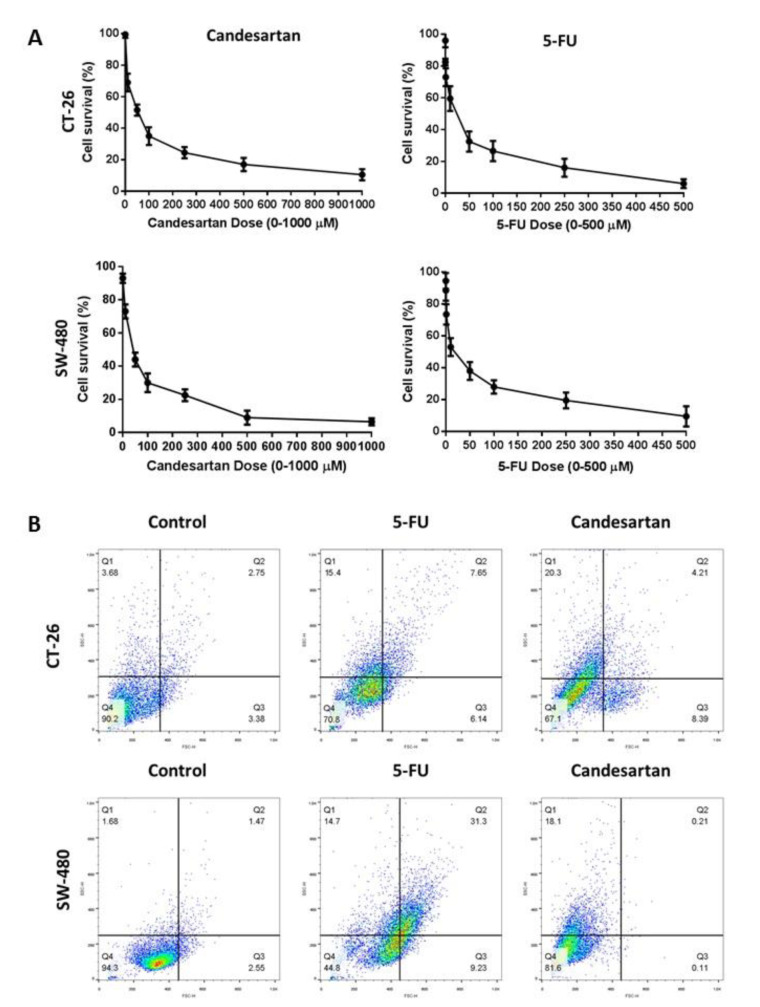
Growth inhibitory and cytotoxic effects of Candesartan on CRC cells. (A) SW-480 and CT-26 cells were treated with different concentrations of Candesartan for 24 and the cell viability was assessed by MTT assay. (B) SW-480 and CT-26 cells were treated with Candesartan for 24 hours and the early and late apoptosis were detected by flow cytometry method. All data was obtained from three independent experiments.

To investigate the cytotoxic effects of candesartan in CRC, the CT-26 and SW-480 cells were treated with different concentrations (0-1000 µM) of candesartan for 24 hours. Our data demonstrated that candesartan inhibited the growth of CRC cells in dose-dependent manner (Figure 1A(Fig. 1)). To further assess the anti-cancer effects of candesartan on CRC, the CT-26 and SW-480 cells were treated with candesartan (20 µM) for 24 hours and apoptosis was determined by flow cytometry method. As shown in Figure 1B(Fig. 1), candesartan significantly induced cell death in CRC cells.

### Candesartan inhibits migratory behaviors of CRC cells

**Figure 2:**
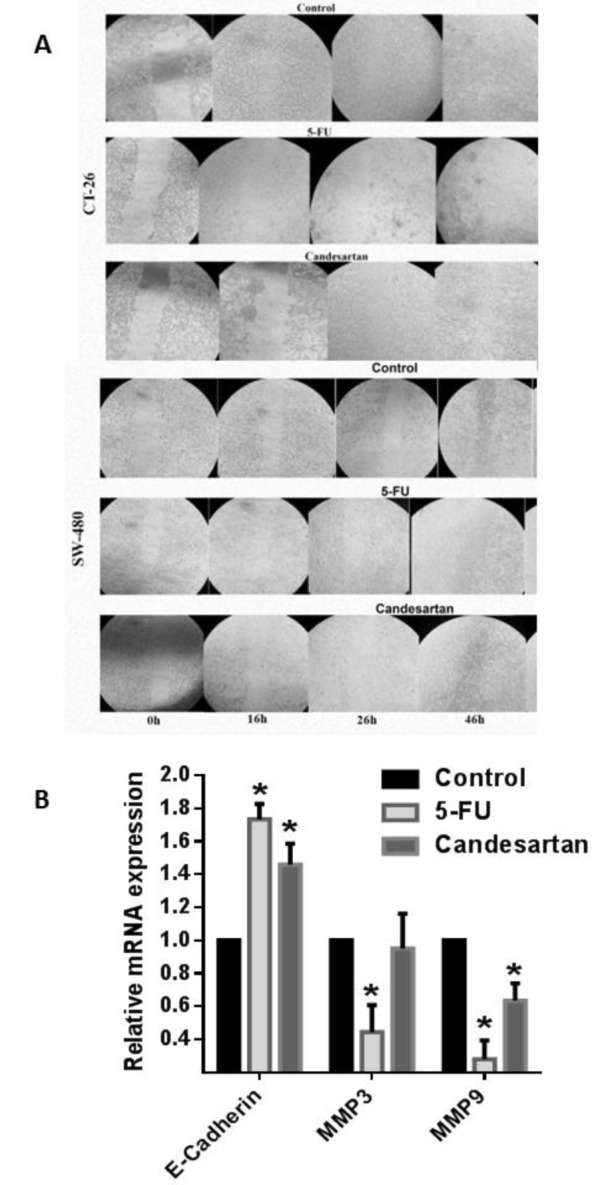
Candesartan inhibits migratory behaviors of CRC cells. (A) Expression level of E-cadherin and MMP3/9 in SW-480 and CT-26 cells in the presence of candesartan was detected by real time RT-PCR. (B) Inhibitory effect of candesartan on the migration of CT-26 cells was measured. All results were obtained from three independent experiments. *p < 0.05.

To identify the regulatory mechanism of candesartan on migratory behaviors of CRC cells, we evaluated the effect of Candesartan on the expression of E-cadherin and matrix metalloproteases-3 and -9. Administration of candesartan decreased the expression of MMP-3 and -9 while significantly increased expression of E-cadherin as a key regulatory factor involved in maintaining cell‐to‐cell adhesion (Figure 2A(Fig. 2)). Consistent with these findings, the migration of candesartan treated CRC cells was significantly inhibited as compared with control group (Figure 2B(Fig. 2)).

### Candesartan suppresses the tumor growth 

**Figure 3:**
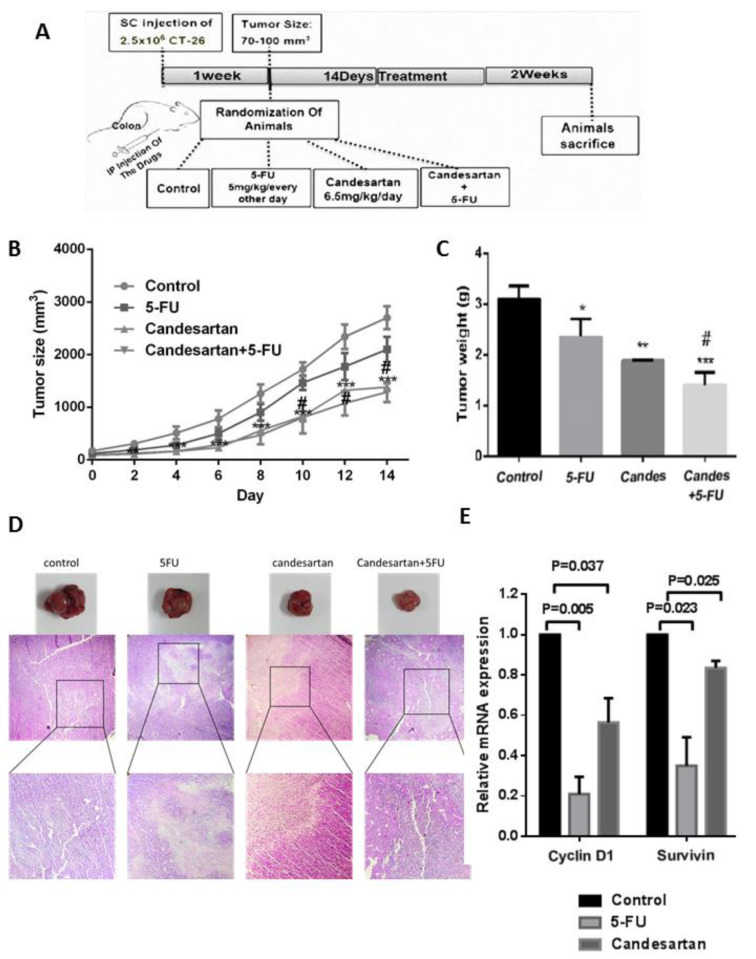
Candesartan suppresses tumor growth in colorectal cancer cells and mouse model (n=6 in each group). (A) Schematic representation of murine tumor model and treatment. (B, C) Tumor size and weight in CRC mouse model treated with Candesartan, 5-FU and their combination. (D) Histological staining of tumor tissues by H&E for evaluating aggregation of tumor cells (T) and necrotic area (N). (E) Expression of cyclin D1 and survivin were measured in CRC tumor tissues using Western blotting. *p < 0.05; **p < 0.01

To further evaluate the regulatory effects of candesartan on colon tumor growth *in vivo*, a mouse model was used in which mice were treated with candesartan either alone or in combination with 5-FU and tumor size and weight were measured and compared between different groups (Figure 3A(Fig. 3)). In accordance with the *in vitro* data, intra-tumoral injection of candesartan reduced the size and weight of tumors in mice (n=6 in each group) (Figure 3B, C(Fig. 3)). Additionally, compared with the 5-FU group, the group receiving adjuvant candesartan demonstrated a more potent anti-cancer effect and the tumor size and weight was lower in the combination group. Interestingly, histological staining of tumor tissues showed higher tissue necrosis in the combination group (Figure 3D(Fig. 3)) suggesting that candesartan synergistically increases the anti-cancer activity of 5-FU in this CRC mouse model. To further determine the anti-proliferative activity of Candesartan, the expression of cyclin D1 and survivin mRNA levels were investigated in CRC tissues. As shown in Figure 3E(Fig. 3), candesartan significantly decreased the expression of cyclin D1 and survivin suggesting the mechanism by which candesartan can inhibit tumor cell proliferation.

### Effects of candesartan on fibrosis

**Figure 4:**
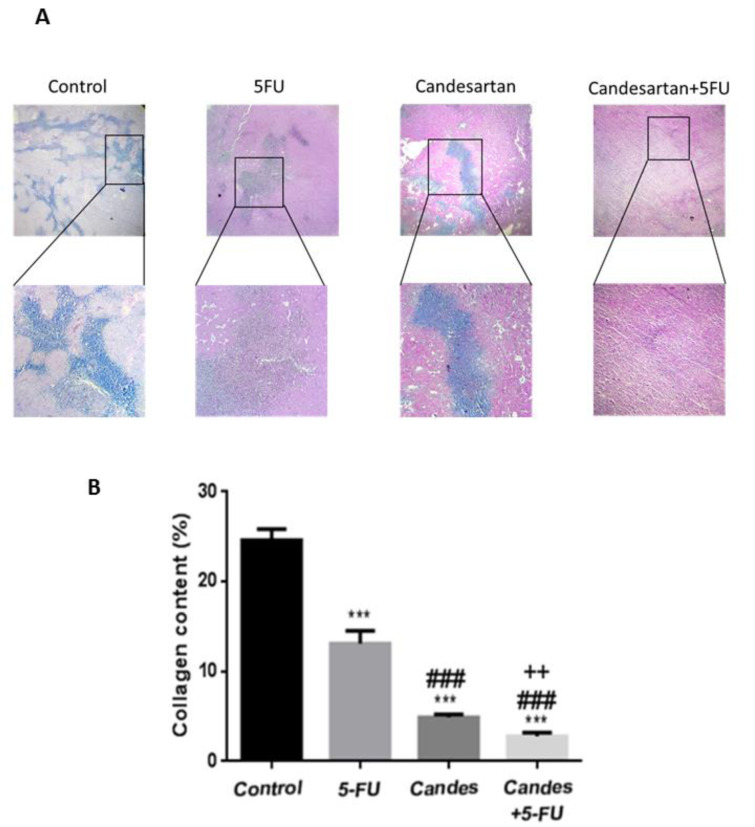
Candesartan suppresses tumor fibrosis in colorectal cancer mouse model (n=6 in each group). (A, B) Trichrome staining for investigating the deposition of collagen fibers in CRC tissues

To explore the regulatory effects of candesartan on fibrosis, the tumor tissues were stained with Masson's trichrome. Colonic fibrosis is characterized by excessive collagen deposition and Histopathological staining findings demonstrated that candesartan significantly inhibits tissue fibrosis and decreased collagen deposition in tumor tissues. Moreover, the tissue fibrosis was also shown to be significantly suppressed in the combination group suggesting that candesartan enhances the anti-fibrosis effects of 5-FU (Figure 4A, B(Fig. 4)).

### Effects of candesartan on oxidant/antioxidant markers

**Figure 5:**
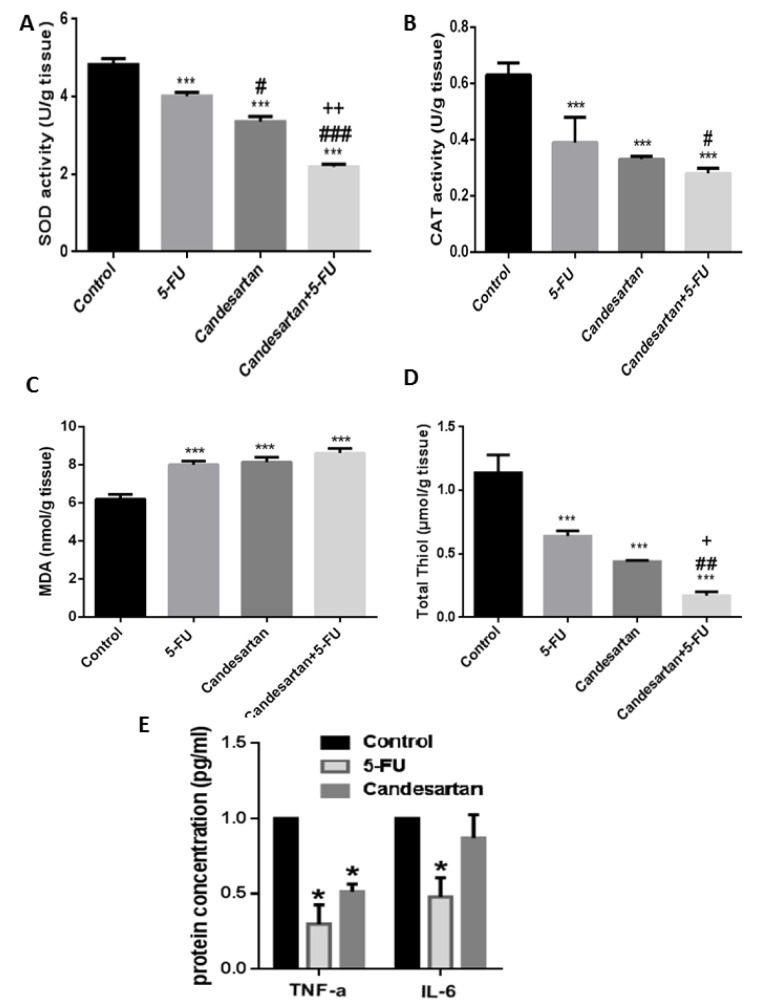
Candesartan induces oxidative stress in homogenized colon samples. The regulatory effect of Candesartan on the enzymatic activity of SOD (A) and catalase (B) and on the MDA (C), total thiol (D) and inflammatory cytokines (E) were investigated in CRC tumor tissues.

To further investigate the role of candesartan on oxidant/antioxidant balance, the levels of SOD, CAT, MDA, and thiol were determined in homogenized colon tissues and results demonstrated in Figure 5A‐D(Fig. 5). The MDA level as a biomarker of oxidative stress increased in treated groups while the enzymatic activity of SOD and CAT and thiol concentrations were significantly decreased compared with the control group. To further confirm that candesartan exerted regulatory effects on oxidant/antioxidant balance, we evaluated the protein levels of inflammatory cytokines in colon tissues. As shown in Figure 5E(Fig. 5), candesartan significantly decreased the expression of TNF-α and Il-6 in comparison with the control group.

### Docking on Candesartan with potential markers associated with RAS pathway

**Figure 6:**
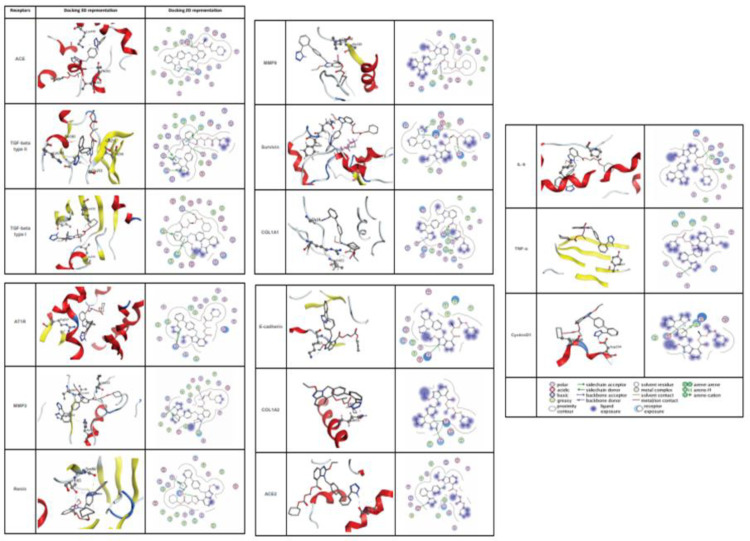
The protein structure in 3D dimensions (left column) and 2D dimensions (right column) related to interaction Candesartan Cilexetil and target proteins

While previous studies have reported that this drug is the inhibitor for angiotensin II type 1 receptor (AT1) (Bregonzio et al., 2008[14]; Baguet et al., 2009[12]; Mengden et al., 2009[31]), we studied the affinity of Candesartan Cilexetil (ligand) and potential markers associated with RAS pathway (Figure 6(Fig. 6)). Docking results are shown that Candesartan could directly interact and inhibit eight proteins as well as AT1 according to Figure 6(Fig. 6) (Score ≤ -7.4 kcal/mol). In particular, ACE had ki = 0.179 µM through two H acceptor bonding among oxygen ligand to oxygen and carbon of THR_302 and THR_301 residues of angiotensin-I-converting enzyme with distance 2.89 and 3.22 respectively and one pi-cation binding among of its 5-ring to nitrogen of LYS_449 with distance 3.89, followed by TGF-beta type II (ki = 0.420 µM; one H acceptor binding of ligand carbonyl group oxygen to carbon of GLY_251 residue with distances 3.52; one H donor binding of ligand carbon to sulfur of CYS_396 residue with distances 3.86; three pi-H binding of ligand (two 5-ring and one 6-ring) to carbon of GLY_253, VAL_ 258, and SER_383 residues with distances 3.71, 3.96, and 3.65 respectively. 

Moreover TGF-beta type I with ki = 0.430 µM; had one H acceptor bonding of ligand carbonyl group oxygen to nitrogen of LYS_232 residue with distances 3.37, and two pi-H binding among its 5-ring and 6-ring to carbon of ILE_211 and LEU_340 residues with distances 3.69 and 3.79. Also, AT1R with ki =0.768 µM; had three H acceptor bonding of ligand nitrogen to nitrogen of ARG_167 residue with distances 3.42, 3.34, and 3.53. Similar data were also detected for MMP3 (ki = 0.796 µM; two H donor binding between carbon and nitrogen of ligand and both of oxygen of LEU_222 residue (distance: 3.29) and nitrogen of HIS_166 residue (distance: 3.49), two H acceptor binding between ligand nitrogen and both of carbon of VAL_163 residue (distance: 3.69) and nitrogen of LEU_164 residue (distance:3.43), one pi-H binding of ligand carbon and 5-ring of HIS_205 residue with distance 4.60. Furthermore, Renin with ki = 0.815 µM had three H acceptor between oxygen of THR_85 residue and nitrogen and oxygen of ligand (distances: 2.99, 2.90 and 3.02), one pi-H binding between of 5-ring of ligand and SER_84 residue with distance 3.85.

The next factor was MMP9 with ki = 1.636 µM with following information: two H acceptor binding of ligand oxygen to nitrogen of LEU_188 and ALA_189 residues with distance 3.26. As shown in Table 2(Tab. 2) and Figure 6(Fig. 6), Survivin with pki = 1.779 µM had one H acceptor binding between carbonyl group oxygen and nitrogen of LYS_15 residue with distance 3.02, one H donor binding of nitrogen to oxygen of GLN_92 residue with distance 2.79, one pi-H between 5-ring of ligand and nitrogen of PHE_93 residue with distance 4.03. Also, ACE2 with ki = 149.862 µM had one H donor binding among nitrogen of ligand to oxygen of ASP_30 residue with distance 3.48.

According to the above-mentioned results, ACE, TGF-beta type I and II, AT1R, MMP3, and Renin, with ki ≤ 0.81 were found to have a strong bond to inhibit Candesartan, due to have lowest inhibition constant between the mentioned drug and these receptors.

See also the Supplementary data.

## Discussion

The therapeutic potential of inhibiting the renin-angiotensin system using AT1R blockers appears to be promising for the management of various cancers including breast, bladder, pancreas, prostate, renal, and colon cancer (Kosugi et al., 2009[28]; Chen et al., 2013[16]; Alhusban et al., 2014[4]; Okazaki et al., 2014[36]; Asgharzadeh et al., 2018[10], 2020[9]). In this study, we used systems and molecular biology to explore the molecular mechanisms of the anti-tumor effects of candesartan in CRC progression in cellular and animal models. Our results suggest that candesartan elicits potent anti-tumor properties by inhibiting cell proliferation, migration, and inducing tumor cell necrosis in CRC.

The renin-angiotensin system is overexpressed in various human cancers which is associated with poor prognosis and aggressive phenotype (Ishikane and Takahashi-Yanaga, 2018[25]; Hashemzehi et al., 2021[24]). Moreover, angiotensin II has the potential to promote tumor cell proliferation and invasion through inducing Wnt/β-catenin signaling pathway (Yu et al., 2016[50]; Li et al., 2018[29]). Cyclin D1 as a key downstream target of Wnt/β-catenin signaling is overexpressed in several human cancers including CRC (Rahmani et al., 2018[42]; Soleimani et al., 2019[47]). Cyclin D1, as a regulatory subunit for cyclin-dependent kinase enzymes, plays an essential role in cell proliferation and cell cycle progression (Peurala et al., 2013[38]). In the present study, we evaluated the anti-tumor effects of candesartan on CRC cells through investigating expression of cyclin D1 and MMPs. Our findings indicated that candesartan treatment inhibits Wnt/β-catenin signaling as visualized by downregulation of cyclin D1, survivin, and MMPs mRNA levels.

Recently, inhibition of Wnt/β-catenin signaling pathway was shown to be related to suppressing tumor cell migration and metastasis (Tong et al., 2016[48]). The MMP enzyme as downstream effectors of Wnt/β-catenin signaling were reported to be involved in cell migration and invasion (Jia et al., 2017[26]; Chen et al., 2021[16]). In line with this data, we illustrated that treatment with candesartan inhibits cancer cell migration. To further confirm the suppressive effects of candesartan on cellular migration the expression levels of E-cadherin and MMP3/9 were investigated and our results indicate that candesartan inhibits migratory behavior of CRC cells by induction of E-cadherin and downregulation of MMP3/9.

Further studies showed that inhibition of Wnt signaling induces tumor cell apoptosis in CRC and breast cancer (Bilir et al., 2013[13]). Wnt/β-catenin signaling was identified as a pro-survival pathway and its inhibition was shown to be involved in cancer cell death and apoptosis (Jia et al., 2019[27]). In agreement with these results, we demonstrated that candesartan induces apoptosis/necrosis in CRC cell lines. 

We also investigated the anti-cancer effects of candesartan in a colon cancer mouse model. Our results demonstrated that candesartan inhibits cancer progression and development through inducing tumor necrosis and changing the oxidant/antioxidant balance in tumor tissue. Results of our study indicate that administration of candesartan increased MDA level and reduced total thiol, SOD, and catalase levels. These results suggest that alterations of oxidant/antioxidant status can be one of the underlying mechanisms contributed to candesartan anti-tumor activities against colon cancer cells. Taken together, we have shown that candesartan elicits its anti-cancer effects by suppressing CRC cell growth and migration, impairing oxidant/antioxidant balance, and enhancing tumor necrosis in CRC cells and tumors, suggesting the therapeutic potential of targeting renin-angiotensin system in colon cancer treatment. Finally, our study demonstrated that candesartan synergistically enhanced the anti-tumor effects of 5-FU suggesting that the AT1R blockers may be used as novel adjuvants in combination with a chemotherapeutic agent in further clinical studies.

## Notes

Ehsan Tabatabai, Majid Khazaei and Amir Avan contributed equally as first authors.

Mohammad Rahmati and Farzad Rahmani (Iranshahr University of Medical Sciences, Iranshahr, Iran; E-mail: Rahmany.farzad@gmail.com) contributed equally as corresponding authors.

## Conflict of interest

The authors have no conflict of interest to disclose.

## Acknowledgements

This research is part of a PhD thesis of Mr. Ehsan Tabatabai, which was supported by the Tabriz University of Medical Sciences and Mashhad University of Medical Sciences.

## Supplementary Material

Supplementary data

## Figures and Tables

**Table 1:**
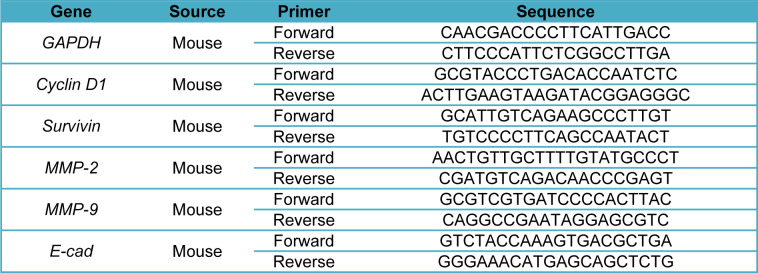
qPCR primer sequences

**Table 2:**
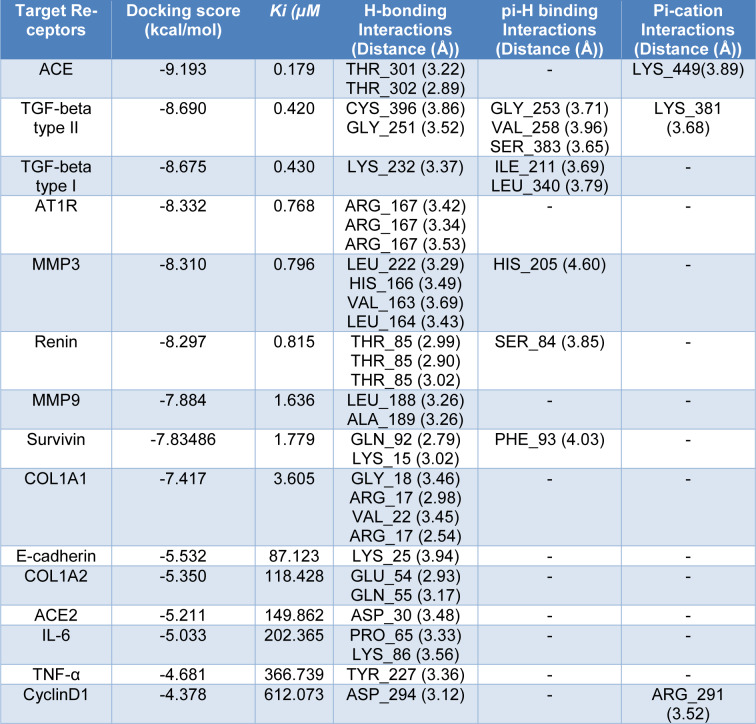
Data for the docking interactions of Candesartan Cilexetil at the active sites of following receptors as the molecular targets.
