# Supplementary material for: Inhibition of angiotensin II type 1 receptor by candesartan reduces tumor growth and ameliorates fibrosis in colorectal cancer
Source: EXCLI J. 2021 May 7;20:863–78. doi: 10.17179/excli2021-3421 (PMC8192880; doi:10.17179/excli2021-3421)
Supplement: Supplementary data [file EXCLI-20-863-s-001.pdf]

EXCLI Journal 2021;20:863-878 – ISSN 1611-2156
Received: January 19, 2021, accepted: April 28, 2021, published: May 07, 2021

Supplementary data to:

Original article:

INHIBITION OF ANGIOTENSIN II TYPE 1 RECEPTOR BY CANDESARTAN REDUCES TUMOR GROWTH AND AMELIORATES FIBROSIS IN COLORECTAL CANCER

Ehsan Tabatabai[1,2#], Majid Khazaei[3,4#], Fereshteh Asgharzadeh[3], Seyedeh Elnaz Nazari[3], Neda Shakour[5,6], Hamid Fiuji[4], Aghigh Ziaeemehr[4], Asma Mostafapour[4], Mohammad Reza Parizadeh[4], Mohammad Nouri[1,2], Seyed Mahdi Hassanian[4,7], Farzin Hadizadeh[5,8], Gordon A Ferns[9], Mohammad Rahmati[1,2*], Farzad Rahmani[10*], Amir Avan[4,11#]

[1] Stem Cell Research Center, Tabriz University of Medical Sciences, Tabriz, Iran
[2] Department of Clinical Biochemistry, Faculty of Medicine, Tabriz University of Medical Sciences, Tabriz, Iran
[3] Department of Physiology, Faculty of Medicine, Mashhad University of Medical Sciences, Mashhad, Iran
[4] Metabolic Syndrome Research Center, Mashhad University of Medical Sciences, Mashhad, Iran
[5] Department of Medicinal Chemistry, School of Pharmacy, Mashhad University of Medical Sciences, Mashhad, Iran
[6] Student Research Committee, Mashhad University of Medical Sciences, Mashhad, Iran
[7] Department of Medical Biochemistry, Faculty of Medicine, Mashhad University of Medical Sciences, Mashhad, Iran
[8] Biotechnology Research Center, Pharmaceutical Technology Institute, Mashhad University of Medical Sciences, Mashhad, Iran
[9] Brighton & Sussex Medical School, Division of Medical Education, Falmer, Brighton, Sussex BN1 9PH, UK
[10] Iranshahr University of Medical Sciences, Iranshahr, Iran
[11] Medical Genetics Research Center, Mashhad University of Medical Sciences, Mashhad, Iran

[#] These authors contributed equally as first authors.

* **Corresponding authors:** Farzad Rahmani, PhD, Iranshahr University of Medical Sciences, Iranshahr, Iran. E-mail: Rahmany.farzad@gmail.com
Mohammad Rahmati, PhD, Department of Clinical Biochemistry, Faculty of Medicine, Tabriz University of Medical Sciences, Tabriz, Iran. Stem Cell Research Center, Tabriz University of Medical Sciences, Tabriz, Iran E-mail: Rahmatibio@gmail.com

http://dx.doi.org/10.17179/excli2021-3421

This is an Open Access article distributed under the terms of the Creative Commons Attribution License (http://creativecommons.org/licenses/by/4.0/).

EXCLI Journal 2021;20:863-878 – ISSN 1611-2156
Received: January 19, 2021, accepted: April 28, 2021, published: May 07, 2021

Supplementary Table 1: Candesartan inhibited CRC cell viability (Raw data concerning Figure1A)

Cytotoxic effects of Candesartan on CRC cells						
**Candesartan doses (nM)**	CT-26 cell viability			SW-480 cell viability		
**0**	100.00	98.00	95.00	91.00	96.00	95.00
**10**	73.00	65.00	71.00	75.00	70.00	76.00
**50**	54.00	49.00	51.00	47.00	44.00	41.00
**100**	39.00	34.00	31.00	29.00	26.00	34.00
**250**	22.00	27.00	25.00	25.00	20.00	22.00
**500**	20.00	14.00	17.00	6.00	10.00	12.00
**1000**	8.00	10.00	13.00	5.00	8.00	7.00

Supplementary Table 2: 5-FU inhibited CRC cell viability (Raw data concerning Figure1A)

Cytotoxic effects of 5-FU on CRC cells						
**5-FU doses (μM)**	CT-26 cell viability			SW-480 cell viability		
**0**	99.00	93.00	97.00	98.00	91.00	99.00
**0.1**	84.00	81.00	82.00	93.00	84.00	86.00
**1**	77.00	69.00	73.00	78.00	69.00	74.00
**10**	65.00	54.00	61.00	57.00	49.00	48.00
**50**	28.00	37.00	30.00	34.00	42.00	39.00
**100**	22.00	31.00	28.00	25.00	31.00	32.00
**250**	20.00	12.00	21.00	16.00	23.00	25.00
**500**	4.00	8.00	9.00	5.00	14.00	8.00

Supplementary Table 3: Candesartan induced CT-26 cell apoptosis (Raw data concerning Figure1B)

Role of Candesartan on CT-26 cell apoptosis		
**Group**	Early apoptosis	Late apoptosis
**Control**	3.38	2.75
**Control**	0.75	2.12
**5-FU**	6.07	8.49
**5-FU**	5.14	7.65
**Candesartan**	9.53	6.09
**Candesartan**	8.39	4.21

Supplementary Table 4: Candesartan induced SW-480 cell apoptosis (Raw data concerning Figure1B)

Role of Candesartan on SW-480 cell apoptosis		
**Group**	Early apoptosis	Late apoptosis
**Control**	2.55	1.47
**Control**	2.28	0.71
**5-FU**	8.46	27.39
**5-FU**	9.23	31.3
**Candesartan**	0.11	0.21
**Candesartan**	3.68	1.44

EXCLI Journal 2021;20:863-878 – ISSN 1611-2156
Received: January 19, 2021, accepted: April 28, 2021, published: May 07, 2021

Supplementary Table 5: The effect of Candesartan in E-cadherin expression in mRNA levels (Raw data concerning Figure 2B)

Group	E-cadherin mRNA levels		
**Control**	1	1	1
**5-FU**	1.8	1.67	1.84
**Candesartan**	1.37	1.55	1.49

Supplementary Table 6: The effect of Candesartan in MMP3 expression in mRNA levels (Raw data concerning Figure 2B)

Group	MMP3 mRNA levels		
**Control**	1	1	1
**5-FU**	0.33	0.56	0.49
**Candesartan**	1.1	0.8	0.92

Supplementary Table 7: The effect of Candesartan in MMP9 expression in mRNA levels (Raw data concerning Figure 2B)

Group	MMP9 mRNA levels		
**Control**	1	1	1
**5-FU**	0.36	0.2	0.39
**Candesartan**	0.56	0.71	0.58

Supplementary Table 8: Candesartan reduced tumor growth in xenograft model (Raw data concerning Figure 3B)

Group	Tumor size (mm^3)							
	Day 0	Day 2	Day 4	Day 6	Day 8	Day 10	Day 12	Day 14
**Control**	140.13	309.57	530.01	851.73	1474.79	1831.54	3240.00	3528.00
**Control**	123.76	193.21	223.51	449.64	856.24	1275.57	1493.00	1752.00
**Control**	126.10	181.50	457.40	524.37	845.37	1832.08	3283.56	3414.32
**Control**	135.11	195.35	455.30	560.79	1862.91	1754.44	2501.00	3190.84
**Control**	327.20	645.39	1274.78	1798.72	1900.28	2001.20	2156.32	2366.44
**Control**	380.72	772.09	1139.95	1588.84	1921.10	2412.01	2912.00	3396.72
**5-FU**	115.34	141.45	292.39	548.87	804.94	1611.06	1000.00	1590.94
**5-FU**	118.52	210.90	274.86	303.49	585.00	862.49	873.98	1172.41
**5-FU**	115.54	151.23	236.69	443.45	1106.21	1730.56	2148.10	2355.43
**5-FU**	107.43	161.88	251.23	437.77	975.78	1588.15	2802.30	3256.19
**5-FU**	135.21	119.19	149.46	262.92	568.92	1361.70	1731.68	1897.21
**5-FU**	167.26	401.33	572.27	1228.09	1889.29	2123.00	2684.41	2721.25
**Candesartan**	70.19	93.41	84.15	188.69	711.00	1020.00	1290.00	1305.95
**Candesartan**	95.72	120.46	144.83	176.33	765.00	1120.00	1360.00	1401.00
**Candesartan**	88.06	81.45	183.18	252.45	505.66	709.91	1353.42	1525.13
**Candesartan**	99.50	129.35	226.74	304.13	451.34	754.74	1310.44	1310.00
**Candesartan**	98.00	127.00	201.00	298.00	501.00	810.00	1305.00	1401.00
**Candesartan**	87.00	131.00	199.00	302.00	498.00	801.00	1320.00	1430.00
**Candesar-tan+5-FU**	96.24	117.95	121.29	281.69	239.39	742.94	1024.67	1228.81
**Candesar-tan+5-FU**	97.15	111.73	205.97	307.37	808.70	1357.67	1500.00	1646.00
**Candesar-tan+5-FU**	102.06	157.42	227.72	276.30	359.51	309.83	701.84	987.74
**Candesar-tan+5-FU**	81.30	114.56	115.00	265.00	376.00	687.00	1201.00	1198.00
**Candesar-tan+5-FU**	87.00	132.00	154.00	281.69	399.00	742.94	1021.00	1321.00
**Candesar-tan+5-FU**	99.00	139.00	231.00	276.30	299.00	310.00	720.00	1001.00

EXCLI Journal 2021;20:863-878 – ISSN 1611-2156
Received: January 19, 2021, accepted: April 28, 2021, published: May 07, 2021

Supplementary Table 9: Candesartan reduced tumor weight in xenograft model (Raw data concerning Figure 3C)

Group	Tumor weight (g)
**Control**	3.82
**Control**	1.97
**Control**	4.07
**Control**	3.48
**Control**	2.90
**Control**	3.60
**5-FU**	1.80
**5-FU**	1.28
**5-FU**	2.17
**5-FU**	4.23
**5-FU**	1.95
**5-FU**	3.41
**Candesartan**	1.89
**Candesartan**	1.89
**Candesartan**	1.91
**Candesartan**	1.89
**Candesartan**	1.88
**Candesartan**	1.96
**Candesartan+5-FU**	1.93
**Candesartan+5-FU**	1.23
**Candesartan+5-FU**	1.09
**Candesartan+5-FU**	1.42
**Candesartan+5-FU**	1.62
**Candesartan+5-FU**	1.23

Supplementary Table 10: The effect of Candesartan in Cyclin D1 expression in mRNA levels (Raw data concerning Figure 3E)

Group	Cyclin D1 mRNA levels		
**Control**	1	1	1
**5-FU**	0.15	0.27	0.33
**Candesartan**	0.65	0.48	0.61

Supplementary Table 11: The effect of Candesartan in Survivin expression in mRNA levels (Raw data concerning Figure 3E)

Group	Survivin mRNA levels		
**Control**	1	1	1
**5-FU**	0.25	0.45	0.3
**Candesartan**	0.86	0.81	0.79

EXCLI Journal 2021;20:863-878 – ISSN 1611-2156
Received: January 19, 2021, accepted: April 28, 2021, published: May 07, 2021

Supplementary Table 12: The effect of Candesartan in Collagen content in tumor tissues (Raw data concerning Figure 4B)

Group	Collagen content
**Control**	25.00
**Control**	20.00
**Control**	18.00
**Control**	28.00
**Control**	31.00
**Control**	25.00
**5-FU**	15.00
**5-FU**	13.00
**5-FU**	14.00
**5-FU**	10.00
**5-FU**	11.00
**5-FU**	14.00
**Candesartan**	7.00
**Candesartan**	5.00
**Candesartan**	6.00
**Candesartan**	6.00
**Candesartan**	4.00
**Candesartan**	5.00
**Candesartan+5-FU**	3.00
**Candesartan+5-FU**	2.00
**Candesartan+5-FU**	4.00
**Candesartan+5-FU**	3.00
**Candesartan+5-FU**	2.00
**Candesartan+5-FU**	5.00

Supplementary Table 13: The effect of Candesartan on the enzymatic activity of SOD, catalase, MDA, and total thiol in tumor tissues (Raw data concerning Figure 5A-D)

Group	SOD activity (U/g tissue)	CAT activity (U/g tissue)	MDA (nmol/g tissue)	Thiol (µmol/g tissue)
**Control**	5.96	.80	5.08	.98
**Control**	4.26	.55	6.73	1.69
**Control**	4.53	.50	6.41	1.01
**Control**	4.87	.59	6.67	.91
**Control**	4.45	.57	5.51	.97
**Control**	5.62	.67	5.13	1.58
**5-FU**	4.00	.35	8.85	.89
**5-FU**	4.18	.41	7.05	.54
**5-FU**	4.53	.40	7.56	.49
**5-FU**	3.50	.41	8.33	.76
**5-FU**	3.96	.39	8.27	.71
**5-FU**	4.03	.42	7.56	.54
**Candesartan**	3.62	.31	8.65	.44
**Candesartan**	3.17	.35	7.95	.47
**Candesartan**	3.31	.33	7.82	.42
**Candesartan**	3.10	.32	8.10	.40
**Candesartan**	3.30	.36	8.30	.44
**Candesartan**	3.20	.29	8.20	.43
**Candesartan+5-FU**	2.34	.25	9.04	.11
**Candesartan+5-FU**	2.18	.31	8.14	.19
**Candesartan+5-FU**	2.08	.29	8.65	.24
**Candesartan+5-FU**	1.90	.28	8.40	.20
**Candesartan+5-FU**	2.10	.27	8.30	.13
**Candesartan+5-FU**	2.00	.27	8.33	.14

EXCLI Journal 2021;20:863-878 – ISSN 1611-2156
Received: January 19, 2021, accepted: April 28, 2021, published: May 07, 2021

Supplementary Table 14: The effect of Candesartan in inflammatory cytokines in tumor tissues (Raw data concerning Figure 5E)

Group	TNF-α (pg/ml)		
**Control**	1	1	1
**5-FU**	0.4	0.21	0.29
**Candesartan**	0.55	0.48	0.52

Supplementary Table 15: The effect of Candesartan in inflammatory cytokines in tumor tissues (Raw data concerning Figure 5E)

Group	IL-6 (pg/ml)		
**Control**	1	1	1
**5-FU**	0.39	0.57	0.51
**Candesartan**	0.76	0.98	0.9
